# At-home disposal practices of used insulin needles among patients with diabetes in China: A single-center, cross-sectional study

**DOI:** 10.3389/fpubh.2022.1027514

**Published:** 2022-12-08

**Authors:** Haixia Tu, Xueqin Lu, Jialu Wang, Zhiqiong Sheng, Danman Liu, Jufang Li, Caixia Sun, Zhiqin Yin

**Affiliations:** ^1^School of Nursing, Wenzhou Medical University, Wenzhou, China; ^2^Endocrinology Department, First Affiliated Hospital of Wenzhou Medical University, Wenzhou, Zhejiang, China; ^3^Nursing Department, First Affiliated Hospital of Wenzhou Medical University, Wenzhou, Zhejiang, China

**Keywords:** diabetes, patients, sharps, needles, disposal

## Abstract

**Background:**

Most insulin injections for people with diabetes are administered at home, thus generating many used needles. Unsafe disposal of these at-home needles can lead to needle stick injuries, blood-borne disease transmission, and environmental contamination. Previous studies have shown varying results on the prevalence of and factors associated with safe sharps disposal practices of people with diabetes.

**Objective:**

To assess the prevalence of and the factors associated with the safe disposal of used insulin needles among patients with diabetes.

**Methods:**

We collected data from 271 insulin-using patients at a tertiary care hospital in China. A self-designed instrument was used to assess sociodemographic data, disease- and treatment-related characteristics, sharps disposal practices, education on diabetes self-management and sharps disposal, and awareness of the potential risks associated with unsafe sharps disposal. Multivariate logistic regression analysis was used to explore factors associated with safe sharps disposal practices.

**Results:**

Only 10.3% (28/271) of participants disposed of used at-home insulin needles in a safe manner, and 14.8% (45/271) of participants had received previous instruction on sharps disposal. Previous sharps disposal instruction (AOR = 4.143, 95% CI = 1.642–10.450) and awareness of the risk of blood-borne pathogen transmission (AOR = 3.064, 95% CI = 1.332–7.046) were associated with safe disposal of used insulin needles.

**Conclusion:**

In our study, the prevalence of safe sharps disposal practices was low, and a minority of respondents had received previous instruction on sharps disposal. Participants who had previously received instruction and were aware of the risk of blood-borne pathogen transmission were more likely to handle sharps safely. Our study findings suggest that health care professionals should pay attention to sharps disposal practices of patients with diabetes and conduct diabetes education programs that include information on safe sharps disposal methods and potential hazards of unsafe sharps disposal.

## Introduction

In 2021, the number of people with diabetes worldwide was estimated to be 537 million (1). Diabetes can cause a variety of complications that lead to stroke, kidney failure, blindness, and lower limb amputation, resulting in reduced quality of life and increased healthcare costs for patients. Moreover, in 2021, an estimated 6.7 million adult deaths were due to diabetes or its complications (1). Therefore, the prevention and control of diabetes is a global public health priority.

In 2021, China had ~141 million people with diabetes, the largest diabetic population in the world; furthermore, by 2045, China is expected to have 175 million patients with diabetes (1). China has made many efforts to manage diabetes (2, 3); however, the continued increase in the number of people with diabetes still poses a significant challenge.

Insulin injections are an important means to control blood glucose and reduce the risk of diabetic complications (4, 5). In a previous study, the number of patients with diabetes using insulin in China was ~9 million (6). Most of these insulin injections are administered at home, thus generating many medical sharps, such as used needles, in the community.

Used needles are classified as infectious and injurious waste. Under no circumstances should these sharps be disposed of in public trash cans or through public waste disposal systems (7). However, China's current regulations for medical waste disposal apply only to medical institutions, not to households or communities (8). Previous studies reported that many patients with diabetes discarded used insulin needles in their household garbage (9–11). Such unsafe disposal can lead to needle stick injuries, blood-borne disease transmission, and environmental contamination. Therefore, there is an urgent need to correct the unsafe handling of medical sharps among people with diabetes in the home environment.

Several factors have been associated with at-home sharps disposal practices of people with diabetes. Previous studies have shown that patients with diabetes who had received education on safe sharps disposal were more likely to safely dispose of at-home sharps than those who had not (12–15). In addition, several studies showed that such education helps improve sharps disposal practices among community-based patients with diabetes (16–19). Additionally, two studies indicated that patients with higher levels of education were more aware of the dangers associated with sharps and the need for safe sharps disposal (20, 21). A study conducted in the United States showed that patients who had diabetes for more than 30 years had the lowest rates of proper sharps disposal (13). Another study indicated that patients who handled needles unsafely had diabetes longer than those who handled sharps safely (22). Moreover, a study in the Philippines revealed that patients with diabetes who had been on insulin for a longer period were less likely to dispose of sharps correctly than those who had been on insulin for a shorter period (23). Another study showed that sharps disposal practices were worse in patients with diabetes who had been receiving insulin for more than a year than those who had been receiving it for less than a year (12). In addition, participants who were aware of the risk of blood-borne pathogen transmission *via* used needles were less likely to dispose of sharps in their household trash (24).

Understanding the current disposal practices for at-home insulin needles among patients with diabetes and identifying the factors associated with safe sharps disposal are essential for tailoring interventions to improve such practices in China. Therefore, the objectives of this study were to (a) assess the prevalence of safe disposal of used insulin needles and (b) explore the factors associated with safe sharps disposal practices within our study group.

## Methods and measurement

### Study design

This was a cross-sectional study using convenience sampling to explore the prevalence of and factors associated with the safe disposal of used insulin needles among Chinese patients with diabetes.

### Participants

We conducted our study from December 2021 to May 2022 in the endocrinology ward of the First Affiliated Hospital of Wenzhou Medical University, a tertiary care hospital in Wenzhou. During the investigation period, patients with diabetes admitted to the ward were invited to participate in our study. Our inclusion criteria were as follows: diabetes mellitus diagnosis, age ≥18 years, insulin use for at least 6 months prior to the survey, insulin injected by the patient himself, and ability to communicate with the investigator. Patients using insulin pumps or diagnosed with gestational diabetes were excluded. Informed consent was obtained, and participation was voluntary. The required sample size was determined based on the following single population proportion formula: $n=\frac{Z_{\alpha /2}^{2}P\left(1-P\right)}{\delta^{2}}.$ Additionally, it was based on the following assumptions: an estimated 18.8% prevalence of safe disposal of used insulin needles (9), a critical value of 95% confidence interval, a 5% margin of error, and a response invalidity rate of 10%. The required sample size was calculated to be 261 and 271 participants ultimately provided valid responses to our survey.

### Survey instrument

For our study, sharps was defined as used insulin needles. Furthermore, safe sharps disposal was defined as discarding used insulin needles in designated sharps or puncture-resistant containers; other sharps disposal methods were categorized as unsafe.

After reviewing the literature, we listed possible factors associated with safe sharps disposal and subsequently developed a five-part tool to collect data based on these factors. The first part collected the following sociodemographic data: age, gender, education, marital status, and presence of children under 14 years of age in the household. Second, we surveyed the following disease and treatment characteristics: duration of diabetes diagnosis, duration of insulin use, schedule of daily insulin injections, needle reuse, and needle stick injuries experienced by the patient or family members. The third part evaluated the participants' disposal method of used needles; in addition, participants with safe sharps disposal practices were required to report how they eventually disposed of containers filled with used needles. Fourth, we assessed whether or not participants had received education on diabetes self-management and sharps disposal. Those who had previously received instruction on sharps disposal were also asked about the source and their primary means of obtaining it; moreover, all participants were asked about their favorite means of obtaining information on sharps disposal. Finally, the fifth part of our survey asked three questions to assess participants' awareness of the potential hazards of unsafe sharps disposal, including needle stick injuries, blood-borne pathogen transmission, and environmental contamination.

We invited five diabetes experts, including two physicians, two advanced practice nurses, and a diabetes educator, to review our original survey instrument. Based on the experts' recommendations, statements that did not match the conventions of expression were modified. The survey instrument was pilot tested in 30 people with diabetes and was appropriate for our study.

### Data collection

Our investigators obtained a list of patients with diabetes receiving insulin injections through the head nurse of the ward and subsequently interviewed these patients. Prior to the interview, investigators received training on the purpose and content of the study as well as the inclusion and exclusion criteria of the sample. Additionally, participants were informed of the purpose and content of the study, and verbal consent was obtained prior to the survey. The investigator first asked the patients whether they had been injecting insulin for 6 months and whether they self-injected; if either question received a “no,” the interview ended. Next, the investigators asked the remaining questions one by one and recorded the participants' verbal responses.

### Ethical considerations

This study was approved by the Ethics Committee in Clinical Research of the First Affiliated Hospital of Wenzhou Medical University (IRB No. KY2021-180).

### Data analysis

Data analysis was performed using SPSS Statistics 22 (IBM, Armonk, NY, USA). The variable of age did not follow a normal distribution and was expressed as medians and quartiles (P25, P75). Categorical variables were represented as cases (*n*) and percentages (%). In our univariate and multivariate analyses, the dependent variable was defined as whether used insulin needles were disposed of safely, i.e., whether they were placed in a designated sharps or puncture-resistant container. Mann–Whitney *U* and chi-square tests were used for univariate analysis for skewed continuous and categorical variables, respectively. Variables with a significance of *p* < 0.20 were included in multivariate logistic regression models to identify factors influencing safe sharps disposal practices, with the significance level set at *p* < 0.05. Variance inflation factor (VIF) was used to check for multicollinearity, and variables with VIF of <5 were included in the multivariate model. In multivariate logistic regression analysis, variables with a significance of *p* < 0.05 were considered statistically significant. Unadjusted odds ratios (UORs) and adjusted odds ratios (AORs) were used to assess the association between independent and dependent variables.

## Results

### Characteristics of the sample

In this study, 420 patients with diabetes using insulin agreed to participate and were interviewed. Of the 149 excluded patients, 65 had been injecting insulin for <6 months, 79 did not inject the insulin themselves, three used an insulin pump, and two were under the age of 18. Altogether, a total of 271 participants provided valid responses. The age of the participants ranged from 20 to 87 years [63 (55, 70)]. Most participants were married, had diabetes for more than 10 years, and used insulin once or twice a day. Furthermore, we found that 69.0% (187/271) of the participants used needles repeatedly. The sociodemographic, disease and treatment characteristics of the participants are shown in Table 1.

**Table 1:** Univariate analysis of factors associated with safe disposal of insulin needles.

	**Total** **(*N* = 271)**	**Safe disposal** **(*n* = 28)**	**Unsafe disposal** **(*n* = 243)**	**χ^2^/*Z***	***p*-Value**
*N* (%)/median (mode)					
Age (years)	63 (55, 70)	60.5 (47, 68)	63 (56, 70)	−1.667	0.095
**Gender**					
Male	161 (59.4)	17 (60.7)	144 (59.3)	0.022	0.882
Female	110 (40.6)	11 (39.3)	99 (40.7)		
**Education**					
None/primary level	151 (55.7)	12 (42.9)	139 (57.2)	2.094	0.148
Junior high level or above	120 (44.3)	16 (57.1)	104 (42.8)		
**Marital status**					
Married	252 (93.0)	24 (85.7)	228 (93.8)	1.443	0.230
Single/unmarried/divorced/widowed	19 (7.0)	4 (14.3)	15 (6.2)		
**Presence of children under 14 year of age**					
Yes	52 (19.2)	4 (14.3)	48 (19.8)	0.484	0.487
No	219 (80.8)	24 (85.7)	195 (80.2)		
**Duration of diabetes diagnosis (years)**					
≤10	78 (28.8)	9 (32.1)	69 (28.4)	0.172	0.678
>10	193 (71.2)	19 (67.9)	174 (71.6)		
**Duration of insulin use (years)**					
≤5	111 (41.0)	15 (53.6)	96 (39.5)	2.054	0.152
>5	160 (59.0)	13 (46.4)	147 (60.5)		
**Schedule of daily insulin injections**					
Once	67 (24.7)	9 (32.1)	58 (23.9)	1.724	0.422
Twice	96 (35.4)	7 (25.0)	89 (36.6)		
Three times or more	108 (39.9)	12 (42.9)	96 (39.5)		
**Needle reuse**					
Yes	187 (69.0)	14 (50.0)	173 (71.2)	5.272	0.022
No	84 (31.0)	14 (50.0)	70 (28.8)		
**Needle stick injuries experienced by the patient or family members**					
Yes	36 (13.3)	7 (25.0)	29 (11.9)	2.673	0.102
No	235 (86.7)	21 (75.0)	214 (88.1)		
**Previous education on diabetes self-management**					
Yes	137 (50.6)	17 (60.7)	120 (49.4)	1.290	0.256
No	134 (49.4)	11 (39.3)	123 (50.6)		
**Previous instruction on sharps disposal**					
Yes	32 (11.8)	9 (32.1)	23 (9.5)	10.317	0.001
No	239 (88.2)	19 (67.9)	220 (90.5)		
**Awareness of the risk of needle stick injuries from unsafe sharps disposal**					
Yes	118 (43.5)	19 (67.9)	99 (40.7)	7.510	0.006
No	153 (56.5)	9 (32.1)	144 (59.3)		
**Awareness of the risk of blood-borne pathogen transmission from sharps disposal**					
Yes	104 (38.4)	18 (64.3)	86 (35.4)	8.864	0.003
No	167 (61.6)	10 (35.7)	157 (64.6)		
**Awareness of the risk of environmental contamination from unsafe sharps disposal**					
Yes	132 (48.7)	19 (67.9)	113 (46.5)	4.583	0.032
No	139 (51.3)	9 (32.1)	130 (53.5)		

Age was tested to be skewed numeric data, expressed as medians and quartiles (P25, P75) and analyzed by Mann–Whitney U-test; categorical variables were described as cases (n) and percentages (%) and analyzed by Chi-square test; safe sharps disposal was defined as placing sharps into designated sharps or puncture-resistant containers.

### Disposal practices of insulin needles

Table 2 presents the disposal methods of insulin needles used by our participants. Only 28 participants placed their insulin needles in designated sharps or puncture-resistant containers. Thus, the prevalence of safe sharps disposal practices was 10.3% (28/271). Most participants (89.7%, 243/271) disposed of insulin needles with or without caps directly in their household trash. Regarding the disposal of containers filled with used needles, two of the 28 respondents who safely disposed of used needles did not respond; of the remaining 26 participants, 18 discarded their sharps containers in their household trash.

**Table 2:** Participants' disposal practices of insulin needles.

**Variables**	**Frequency**
	**(%)**
**Disposal of insulin needles (*N* = 271)**	
Designated sharps containers	5 (1.8)
Puncture-resistant containers	23 (8.5)
Directly into household garbage with recapping	191 (70.5)
Directly into household garbage without recapping	52 (19.2)
**Disposal of designated or puncture-resistant containers (*n* = 26)**	
Health care facility	4 (15.4)
Handed over to the cleaner	2 (7.7)
Household garbage	18 (69.2)
Placed in her toilet and had not been disposed of yet	1 (3.8)

### Instruction on sharps disposal

Table 3 shows that 14.8% (45/271) of respondents had received previous instruction on how to handle sharps. The most frequently mentioned source of information was a nurse, followed by a doctor, a friend or relative, and others. The information was primarily obtained verbally. Additionally, the majority (69.7%, 189/271) of participants wanted to receive information regarding sharps disposal verbally.

**Table 3:** Instruction on sharps disposal.

**Variables**	**Frequency**
	**(%)**
**Previous instruction on sharps disposal (*N* = 271)**	
Yes	45 (14.8)
No	259 (85.2)
**Source of sharps disposal instruction (*n* = 45)**	
Nurse	24 (75.0)
Doctor	5 (15.6)
Pharmacist	0 (0.0)
Friend or relative	2 (6.3)
Others^a^	1 (3.1)
**Primary means of obtaining information on sharps disposal (*n* = 32)**	
Verbal	26 (81.3)
Booklets, magazines or books	0 (0.0)
Television or broadcast	0 (0.0)
Internet	6 (18.8)
**Favorite means of obtaining information on sharps disposal (*n* = 271)**	
Verbal	189 (69.7)
Booklets, magazines or books	26 (9.6)
Television or broadcast	14 (5.2)
Internet	42 (15.5)

a Others referred to a community worker.

### Univariate analysis of safe sharps disposal practices

After univariate analysis of factors associated with the safe disposal of insulin needles, the following nine variables were entered into the multivariate logistic regression model: age, education, duration of insulin use, needle reuse, needle stick experience, previous instruction on sharps disposal, awareness of the risk of needle stick injuries, awareness of the risk of blood-borne pathogen transmission and awareness of the risk of environmental contamination (*p* < 0.20; Table 1).

### Multivariate analysis of safe sharps disposal practices

The Box–Tidwell test indicated that the relationship between age and safe sharps disposal was linear. The VIF values of the nine independent variables that entered the multivariate logistic regression were < 2 and no collinearity was detected. Hosmer-Lemeshow test [chi-square = 0.014, degrees of freedom (df) = 1, *p* = 0.906] and omnibus tests of model coefficients (chi-square = 16.680, df = 2, *p* = 0.000) suggested that forward stepwise (likelihood ratio) multivariate logistic regression was appropriate.

After adjusting for potential confounders, previous instruction on sharps disposal and awareness of the risk of blood-borne pathogen transmission remained two significant predictors of safe insulin needle disposal practices (Table 4). Patients with diabetes who had previously received sharps disposal instruction were 4.143 times (AOR = 4.143, 95% CI = 1.642–10.450) more likely to dispose of insulin needles safely than those who had not. Furthermore, participants who were aware of the risk of blood-borne pathogen transmission from unsafe sharps disposal were 3.064 times more likely to dispose of needles safely than those who were not (AOR = 3.064; 95% CI, 1.332–7.046).

**Table 4:** Multivariate analysis of factors associated with safe disposal of insulin needles (*N* = 271).

**Variables**	**AOR**	**95% CI**	***p*-Value**
**Previous instruction on sharps disposal**			
Yes	4.143	1.642, 10.450	0.003
No	Reference		
**Awareness of the risk of blood-borne pathogens transmission from sharps disposal**			
Yes	3.064	1.332, 7.046	0.008
No	Reference		

AOR, adjusted odds ratio; CI, confidence interval.

## Discussion

This was a cross-sectional study using a self-administered instrument to gain insight into insulin needle disposal practices among patients with diabetes and to explore factors associated with safe sharps disposal practices.

### Disposal practices of insulin needles

In the present study, the prevalence of safe sharps disposal practices was low, with only 10.3% of participants safely disposing of their insulin needles. This finding is similar to previous studies conducted in China (9, 11) and other countries (14, 15, 20, 25). However, two studies from the United States showed much higher rates of safe sharps disposal, with Montoya and Huang reporting 67 and 59%, respectively (13, 22). This difference may be related to the specific policies and clear guidelines for at-home sharps disposal that exist in the United States (26, 27).

Of the participants who placed used needles in designated sharps or puncture-resistant containers, most ultimately discarded these containers in their household trash. This is in line with findings from Bangladesh (25), Sri Lanka (12), and India (28). This may be due to the lack of sharps collection terminals or community sharps disposal programs; patients with diabetes do not have access to sharps container disposal and instead place them in household waste (12).

### Associated factors of safe sharps disposal

In our study, previous instruction on sharps disposal and awareness of the risk of blood-borne pathogen transmission were two factors significantly associated with safe sharps disposal practices. Our findings showed that patients with diabetes who had received previous sharps disposal instruction were more likely to have safe sharps disposal practices compared to those who had not. This is supported by the results of other cross-sectional studies (12–15). Furthermore, a study conducted in India found that a provider-initiated patient-centered insulin-use health education program with information on sharps disposal significantly improved the knowledge and practice of used insulin sharps disposal among people with diabetes (18). A quasi-experimental study conducted in northeast Peninsular Malaysia reported that a structured community sharps disposal education module that included content on the proper handling of sharps, was effective in improving sharps disposal knowledge and encouraging safe sharps disposal practices among Malaysian patients with diabetes (16). The positive relationship between previous instruction on sharps disposal and safe sharps disposal practices can be explained by the health belief model, where information on safe sharps disposal can serve as a cue for action, activating readiness and stimulating positive health-related behaviors among people with diabetes (29). In contrast, a study conducted in the Philippines showed that sharps disposal instruction from health care professionals was not a significant predictor of safe sharps disposal practices (23). They speculated that this might result from either patients having low adherence to sharps disposal advice from health care professionals or health care professionals providing incorrect information in the first place.

In our study, only a few respondents had previously received instruction on sharps disposal, which is similar to findings from Sri Lanka (12). The reason for this may be that health care professionals and patients are not fully aware that home sharps disposal is a serious public health issue. According to our results, nurses were the primary provider of sharps disposal information, which may result from the fact that education on insulin injection in China is mainly performed by nurses and diabetes educators (30). Notably, most diabetes educators in China are also nurses (31). Future research is needed to identify barriers that prevent other health care professionals from educating patients about safe sharps disposal. Most participants in this study preferred to receive verbal information on how to safely dispose of sharps. Therefore, nurses, diabetes educators, physicians, and pharmacists should provide early and sustainable verbal counseling to their patients to improve sharps disposal practices. Furthermore, online and offline materials, such as pamphlets and videos, should be available for patients to independently review safe sharps disposal practices at home.

Our study also showed that patients with diabetes who were aware of the risk of blood-borne pathogen transmission were more likely to have safe practices than patients who were not aware of this risk. This finding is consistent with a study conducted in Pakistan, which revealed that people with diabetes who knew the risk of blood-borne disease transmission were less likely to dispose of sharps in their household trash (24). Understanding precautions that can reduce the spread of blood-borne pathogens, such as safe sharps disposal, is important (32). This can be explained by the health belief model, whereby patients who have diabetes understand the serious consequences of blood-borne disease transmission and develop fears about their current behavior, which can motivate them to change this behavior (29). In short, when discussing sharps disposal with patients who have diabetes, health care professionals should inform them of the potential harms associated with unsafe disposal.

### Limitations

The present study has some limitations. First, some factors that may influence sharps disposal practices were not included, such as income, place of residence, and comorbid blood-borne diseases. Second, some participants may have had difficulty accurately recalling past events, such as needle stick injuries, duration of diabetes diagnosis, and duration of insulin use, so we cannot exclude the possibility of recall bias. Third, participants in this study were recruited from a single hospital through convenience sampling, which limits the generalizability of our study. A multi-center study with larger samples is needed. In addition, interventional studies could be considered.

## Conclusion

In our hospital-based sample of patients with diabetes, the prevalence of safe sharps disposal was low, and only a minority of respondents had received previous sharps disposal instruction. Patients who had previously received sharps disposal instruction and were aware of the risk of blood-borne pathogen transmission were more likely to perform sharps disposal correctly. Our results may provide a basis for conducting sharps education programs for patients with diabetes and urging the government to issue regulations for the management of sharps disposal in the community. Our study findings suggest that safe sharps disposal methods and potential risks of unsafe sharps disposal should be incorporated into education programs for patients with diabetes and that health care professionals should receive training on the safe disposal of home-generated sharps.

## Data availability statement

The datasets analyzed during the current study are available from the corresponding author on reasonable request.

## Ethics statement

The studies involving human participants were reviewed and approved by the Ethics Committee in Clinical Research of the First Affiliated Hospital of Wenzhou Medical University (IRB No. KY2021-180). The patients/participants provided their written informed consent to participate in this study.

## Author contributions

HT performed the analysis, wrote the initial manuscript, and revised the manuscript. XL coordinated the data collection and interpreted the results. XL and CS reviewed the manuscript. JW, ZS, and DL contributed to data collection and data analysis. JL designed the study. CS supervised data collection. ZY and HT conceptualized and designed the study. ZY and JL reviewed and revised the manuscript. All authors approved the final manuscript as submitted and agreed to be accountable for all aspects of the work.
